# A dynamic assessment of various non-Newtonian models for ternary hybrid nanomaterial involving partially ionized mechanism

**DOI:** 10.1038/s41598-022-14312-9

**Published:** 2022-06-19

**Authors:** Umar Nazir, Muhammad Sohail, Poom Kumam, Kanokwan Sitthithakerngkiet, Abd Allah A. Mousa, Muhammad Jahangir Khan, Ahmed M. Galal

**Affiliations:** 1 Department of Applied Mathematics and Statistics, Institute of Space Technology, P.O. Box 2750, Islamabad, 44000 Pakistan; 2 Department of Mathematics, Khwaja Fareed University of Engineering & Information Technology, Rahim Yar Khan, 64200 Pakistan; 3 Center of Excellence in Theoretical and Computational Science (TaCS-CoE) & KMUTT Fixed Point Research Laboratory, Room SCL 802 Fixed Point Laboratory, Science Laboratory Building, Departments of Mathematics, Faculty of Science, King Mongkut’s University of Technology Thonburi (KMUTT), 126 Pracha-Uthit Road, Bang Mod, Thung Khru, Bangkok, 10140 Thailand; 4 Department of Medical Research, China Medical University Hospital, China Medical University, Taichung, 40402 Taiwan; 5 Intelligent and Nonlinear Dynamic Innovations Research Center, Department of Mathematics, Faculty of Applied Science, King Mongkut’s University of Technology North Bangkok (KMUTNB), 1518, Wongsawang, Bangsue, Bangkok, 10800 Thailand; 6 Department of Mathematics, College of Science, Taif University, P.O. Box 11099, Taif, 21944 Saudi Arabia; 7 Department of Advance Materials and Technologies, Faculty of Materials Engineering, Silesian University of Technology, 44-100 Gliwice, Poland; 8 Mechanical Engineering Department, College of Engineering, Prince Sattam Bin Abdulaziz University, Wadi Addawaser, 11991 Saudi Arabia; 9 Production Engineering and Mechanical Design Department, Faculty of Engineering, Mansoura University, P.O 35516, Mansoura, Egypt

**Keywords:** Mathematics and computing, Nanoscience and technology

## Abstract

The dynamic of fluids and coolants in automobiles are achieved by enhancement in heat energy using ternary hybrid nanostructures. Ternary hybrid nanomaterial is obtained by suspension of three types of nanofluid (aluminum oxide, silicon dioxide and titanium dioxide) in base fluid (EG). Prime investigation is to address comparison study in thermal energy involving various flow models termed as Maxwell fluid and Williamson fluid. This exploration is carried out by partially ionized fluidic particles in the presence of ternary hybrid nanomaterial over cone. Heat transfer is carried out by heat source and thermal radiation. Equations regarding Ordinary differential are achieved from PDEs using variable transformations. The numerical consequences are obtained implementing finite element method. Flow into fluid particles is enhanced versus higher values of Hall and ion slip parameters. Thermal performance as well as flow performance for the case Williamson fluid is better than for case of Maxwell fluid. Production via energy is boosted versus heat source parameter.

## Introduction

Latest insurrection in manufacturing and science machineries has prepared several effects possible which where incredible in past developed of nano-particles is since of modern progress in nano-technology. Technology based on thermal design and development of nano-scale (solid particles) have been initiated approaches regarding synthesis fluids involving nano-metallic are called nano-fluids. Tri-hybrid nanomaterial has higher thermal conductivity as compared hybrid nanofluid and nanoparticles, which are used in several engineering applications which are applicable in engineering process, cancer therapy, hair care products, electrical insulators, green tires, dental products, fuel cells, solar cells, optical chemical sensors, bio-sensors and automotive parts. Additionally, the concept related to electrically conducting fluids is totally distinct as compared concept related to conducting liquids considering absence of magnetic field. Magnetohydrodynamic (MHD) flow is defined as electrically conducting fluids in the occurrence of magnetic field. Such developments regarding modeling are required Ohm’s law and Maxwell’s equations along with conservations laws. Applications of MHD are utilized in nanofluid pumping, magnetic drug targeting, pumping of seawater, cancer tumor treatment and fluid pumping. These studies includes both theoretical and experimental in term nanofluids, hybrid nanoparticles and tri-hybrid nanoparticles. Here, we discuss the related work. Nazir et al.^1^ visualized thermal and solute transportation model in Williamson liquid involving hybridity of nanomaterial over a stretching surface in the presence of Forchheimer theory. They have utilized finite element method to simulate numerical results. Riaz et al.^2^ investigated suspension regarding fluid particles in non-Newtonian model using curved passage. Sadiq et al.^3^ studied thermal transfer model in Maxwell liquid in the occurrence of hybrid nanoparticles via thin film. Sadiq et al.^4^ performed heat energy performance inserting nanofluid using a stretching surface. Pushpa et al.^5^ simulated numerical consequences of nanoparticles in convective flow including heat dissipation in thin baffle. Marzougui et al.^6^ used lid driven cavity to conduct numerical consequences in the presence of entropy generation inserting nanoparticles under magnetic field. Shafiq et al.^7^ discussed fluidic effects in Walters’ B fluid including stagnation point flow in a Riga plate using thermal radiation via statistical method. Swain et al.^8^ studied slip conditions to conduct numerical consequences of hybrid nanoparticles over a porous shrinking sheet under chemical reaction. Imran et al.^9^ simulated study regarding solar collector in mono and hybrid nanomaterial using nanoparticles shapes over flat plate. Imran et al.^10^ performed features of nanofluid in term of bio-convection containing motile microorganisms induced by paraboloid surface. Farooq et al.^11^ computed impacts of entropy generation in nanofluid capturing thermal radiation. Hou et al.^12^ discussed features based on heat transfer mechanism in Pseudo-Plastic material using ternary hybrid nanoparticles towards a heated surface. Wang et al.^13^ developed mathematical approach related mass diffusion as well as heat transfer in the presence of generalized theory involving variable properties. Alhazmi et al.^14^ analyzed modified fluxes in Williamson liquid in term of mass and heat mechanisms. Nazir et al.^15^ discussed heat energy performance in Casson liquid involving nanoparticles using stretching surface. Imran et al.^16^ estimated thermal features of Bioconvection flow in cross liquid involving swimming microorganisms. Safdar et al.^17^ used Buongiorno’s Model in heat transfer model via Maxwell liquid. Naseem et al.^18^ simulated variable thermal conductivity using Soret and Dufour effects in hydro- magnetized flow. Tripathi and Kumari^19^ estimated generalized heat transfer characterizations in thin film using thermocapillary convection. Kumari and Tripathi^20^ discussed features of self-rewetting liquid under the action of gravitational force considering concept of Marangoni convection. Kumar et al.^21^ studied role of magnetic field in brinkman type nanoparticles in the presence of convection flow considering chemical reaction. Kumar et al.^22^ studied characterizations of thermal transport using influences of Soret and second order chemical reaction over a heated vertical plate considering porous medium and thermal radiation. Kumar et al.^23^ discussed the thermal aspects of magnetic parameter into nanoparticles named as CNT in the presence of viscous dissipation. Kumar et al.^24^ estimated numerical consequences of thermal radiation in heat transfer using Williamson rheology considering Joule heating. Kumar et al.^25^ analyzed mathematical modeling of non-Newtonian liquid using concept of magnetic dipole and thermal radiation in the presence of activation energy. Kumar et al.^26^ studied entropy generation in Casson liquid considering influence of magnetic field and activation energy. Kumar et al.^27^ estimated features of entropy generation and magnetic field into nanoparticles past a rotating disk including concept of activation energy^26^.

It is claimed that there is no investigations on tri-hybridity of nanoparticles in ethylene glycol involving two fluid models termed as Maxwell and Williamson fluids past a cone considering ion slip and Hall forces. Additionally, thermal aspects regarding heat generation, heat absorption and thermal radiation are added into heat energy equation. The comparative investigation among Maxwell and Williamson fluids are studied whereas comparison among pure fluid, nanofluid, hybrid nanomaterial and tri-hybrid nanomaterial is also observed. The present analysis is contained into five sections. First section is related to literature review. Mathematical work is prescribed in section two and section three is based on numerical work. Section four is about discussion and results while last section is related to conclusions.

## Descriptions regarding modeling and novelty

**Figure 1:**
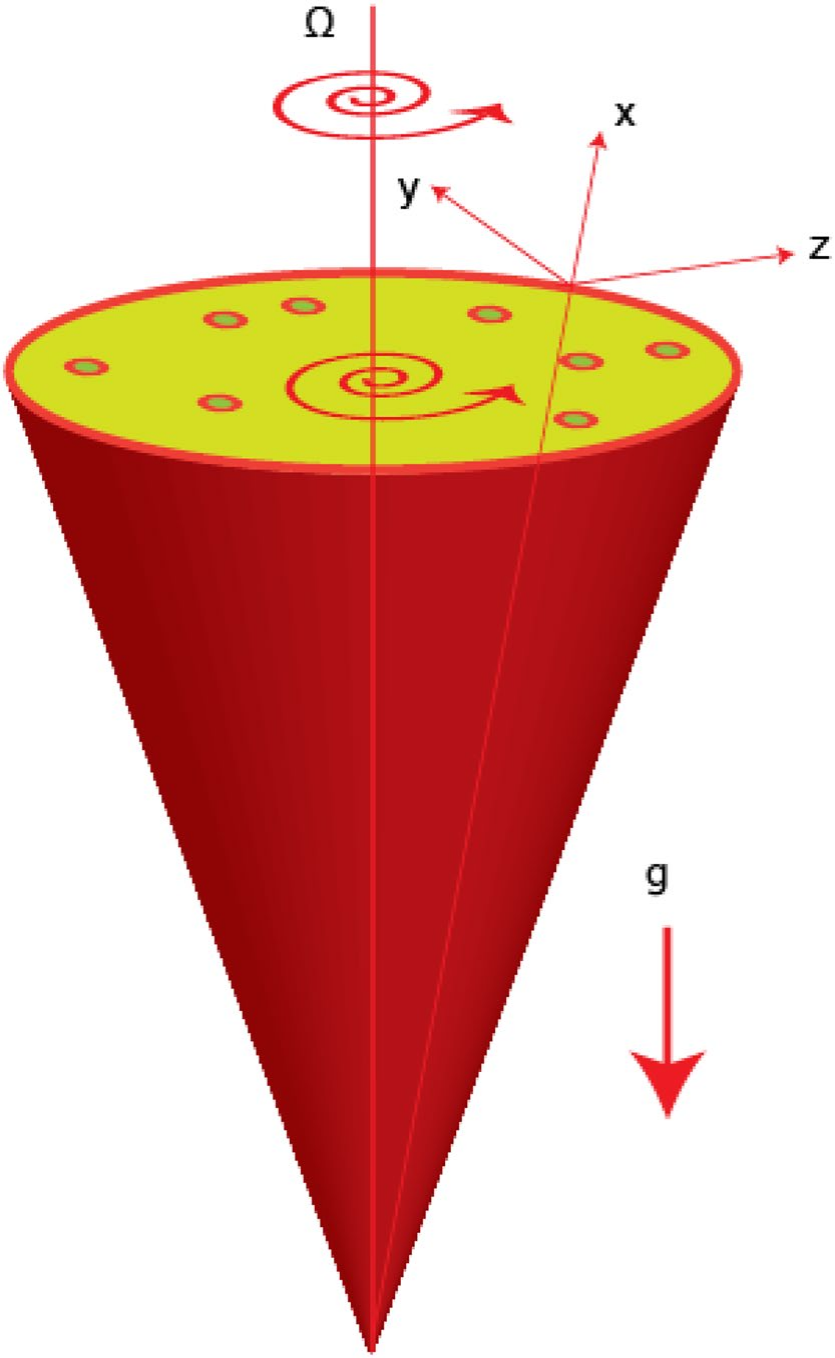
Geometry of developed analysis.

Thermal achievement in 3D Williamson and Maxwell liquids is visualized past a cone with the suspension of tri-hybrid nanomaterial in base liquid termed as ethylene glycol. Fluidic motion is produced with help of angular velocity of cone. Heat energy is taken place using thermal features thermal radiation, Joule heating and heat source. Moreover, fluid is considered as viscous in rotating disk. Figure 1 depicts the flow configuration and thermal properties regarding tri-hybrid nanomaterial is prescribed in Table 1.

**Table 1:** Nanoaprticles properties in term of thermal in EG^13^.

	$K$	$\rho$	$\sigma$
${Al}_{2}{O}_{3}$	32.9	6310	$5.96\times {10}^{7}$
$Si{O}_{2}$	1.4013	2270	$3.5\times {10}^{6}$
$Ti{O}_{2}$	8.953	4250	$2.4\times {10}^{6}$
EG	0.144	884	$0.125\times {10}^{-11}$

Conservations laws in term of momentum and energy^28^^,^^30^ for non-Newtonian fluids flow are

1 $${\mathbf{\nabla }} \cdot {\varvec{V}} = 0$$

2 $$\rho_{Thnf} \frac{{{\varvec{dV}}}}{{{\varvec{dt}}}} = - \nabla {\text{P}} + {\mathbf{J}} \times {\mathbf{B}} + \nabla \cdot {\varvec{\tau}},$$

3 $$\nabla \times {\mathbf{E}} = \frac{{\partial {\varvec{B}}}}{\partial t},\user2{ }\mu_{Thnf} {\varvec{J}} = \nabla \times {\mathbf{B}}, \nabla \cdot {\varvec{B}} = 0,\user2{ }$$

4 $${\varvec{J}} = {\varvec{\sigma}}_{{{\varvec{Thnf}}}} \left[ {{\varvec{E}} + {\varvec{V}} \times {\mathbf{B}}} \right] - \frac{{\beta_{e} }}{{\left| {\varvec{B}} \right|}}\left( {{\varvec{J}} \times {\varvec{B}}} \right) + \frac{{\beta_{i} \beta_{e} }}{{\left| {\varvec{B}} \right|^{2} }}\left( {{\varvec{J}} \times {\varvec{B}}} \right) \times {\varvec{B}},$$

5 $$\left( {\rho C_{p} } \right)_{thnf} \frac{{{\varvec{d}}T}}{{{\varvec{dt}}}} = K_{Thnf} \nabla^{2} T - \nabla \cdot {\varvec{q}} + Q_{0} \left( {T - T_{\infty } } \right).$$

The reduced form of PDEs^13^^,^^28^^,^^30^ are achieved using BLA (boundary layer approximations) which are formulated as

6 $$\frac{{\partial \left( {XU} \right)}}{\partial X} + \frac{{\partial \left( {XV} \right)}}{\partial Z} = 0,$$

7 $$\begin{gathered} U\frac{\partial U}{{\partial X}} + W\frac{\partial U}{{\partial Z}} = \frac{{\left( {B_{0} } \right)^{2} \sigma_{Thnf} }}{{\rho_{Thnf} \left[ {\left( {1 + \beta_{e} \beta_{i} } \right)^{2} + \left( {\beta_{e} } \right)^{2} } \right]}}\left[ {V\beta_{e} - \left( {1 + \beta_{i} \beta_{e} } \right)U} \right], \hfill \\ + \frac{{V^{2} }}{X} + G\beta \left( {T - T_{\infty } } \right)\cos \alpha + \nu_{thnf} \frac{{\partial^{2} U}}{{\partial Z^{2} }} + \sqrt 2 \left( {\nu_{thnf} } \right){\Gamma }\frac{\partial U}{{\partial Z}}\frac{{\partial^{2} U}}{{\partial Z^{2} }} \hfill \\ - \lambda_{1} \left[ {U^{2} \frac{{\partial^{2} U}}{{\partial X^{2} }} + V^{2} \frac{{\partial^{2} U}}{{\partial Z^{2} }} + 2UV\frac{{\partial^{2} U}}{\partial Z\partial X}} \right], \hfill \\ \end{gathered}$$

8 $$- \frac{UV}{X} - \lambda_{1} \left[ {U^{2} \frac{{\partial^{2} V}}{{\partial X^{2} }} + V^{2} \frac{{\partial^{2} V}}{{\partial Z^{2} }} + 2UV\frac{{\partial^{2} V}}{\partial Z\partial X}} \right] + \nu_{thnf} \frac{{\partial^{2} V}}{{\partial Z^{2} }} + \sqrt 2 \left( {\nu_{thnf} } \right){\Gamma }\frac{\partial V}{{\partial Z}}\frac{{\partial^{2} V}}{{\partial Z^{2} }},$$

9 $$U\frac{\partial T}{{\partial X}} + W\frac{\partial T}{{\partial Z}} = \frac{{K_{thnf} }}{{\left( {\rho C_{p} } \right)_{thnf} }}\frac{{\partial^{2} T}}{{\partial Z^{2} }} + \frac{{Q_{0} }}{{\left( {\rho C_{p} } \right)_{thnf} }}\left( {T - T_{\infty } } \right) + \frac{{\sigma^{*} 16T_{0}^{3} }}{{3K^{*} \left( {\rho C_{p} } \right)_{thnf} }}\frac{{\partial^{2} T}}{{\partial Z^{2} }}.$$

The boundary conditions^28^ are derived as

10 $$U = 0, V = {\Omega }X\sin \alpha , T = T_{W} :Z = 0, U \to 0, V \to 0,T \to T_{\infty } :Z \to \infty .$$

Thermal correlations in term of three types of nanoparticles in base fluid^13^ are defined as

11 $$\rho_{Thnf} = \left( {1 - phi_{1} } \right)\left\{ {\left( {1 - phi_{2} } \right)\left[ {\left( {1 - phi_{3} } \right)\rho_{f} + phi_{3} \rho_{3} } \right] + phi_{2} \rho_{2} } \right\} + phi_{1} \rho_{1,}$$

Thermal correlations in term of three types of nanoparticles in base fluid^13^ are defined as

12 $$\rho_{Thnf} = \left( {1 - phi_{1} } \right)\left\{ {\left( {1 - phi_{2} } \right)\left[ {\left( {1 - phi_{3} } \right)\rho_{f} + phi_{3} \rho_{3} } \right] + phi_{2} \rho_{2} } \right\} + phi_{1} \rho_{1,}$$

13 $$\frac{{K_{Thnf} }}{{K_{hnf} }} = \frac{{K_{1} + 2K_{hnf} - 2phi_{1} \left( {K_{hnf} - K_{1} } \right)}}{{K_{1} + 2K_{hnf} + phi_{1} \left( {K_{hnf} - K_{1} } \right)}}, \frac{{K_{nf} }}{{K_{f} }} = \frac{{K_{3} + 2K_{f} - 2phi_{3} \left( {K_{f} - K_{3} } \right)}}{{K_{3} + 2K_{f} + phi_{3} \left( {K_{f} - K_{3} } \right)}},$$

14 $$\frac{{\sigma_{Tnf} }}{{\sigma_{hnf} }} = \frac{{\sigma_{1} \left( {1 + 2phi_{1} } \right) - phi_{hnf} \left( {1 - 2phi_{1} } \right)}}{{\sigma_{1} \left( {1 - phi_{1} } \right) + \sigma_{hnf} \left( {1 + phi_{1} } \right)}}, \frac{{\sigma_{hnf} }}{{\sigma_{nf} }} = \frac{{\sigma_{2} \left( {1 + 2phi_{2} } \right) + \varphi_{nf} \left( {1 - 2phi_{2} } \right)}}{{\sigma_{2} \left( {1 - phi_{2} } \right) + \sigma_{nf} \left( {1 + phi_{2} } \right)}},$$

15 $$\frac{{\sigma_{nf} }}{{\sigma_{f} }} = \frac{{\sigma_{3} \left( {1 + 2phi_{3} } \right) + phi_{f} \left( {1 - 2phi_{3} } \right)}}{{\sigma_{3} \left( {1 - phi_{3} } \right) + \sigma_{f} \left( {1 + phi_{3} } \right)}}.$$

Boundary conditions^28^^,^^30^ are derived as

16 $$U = - \frac{{{\Omega }X\sin \alpha F^{\prime } }}{2}, V = {\Omega }X\sin \alpha G, W = \left( {{\Omega }\nu_{f} \sin \alpha } \right)^{\frac{1}{2}} F, {\Theta } = \frac{{T - T_{\infty } }}{{T_{w} - T_{\infty } }},\eta = \left( {\frac{{{\Omega }\sin \alpha }}{{\nu_{f} }}} \right)^{\frac{1}{2}} Z.$$

Dimensionless ODEs^28^^,^^30^ are

17 $$F^{\prime \prime \prime } - \beta \left( {F^{2} F^{\prime \prime \prime } - 2FF^{\prime } F^{\prime \prime } } \right) + \lambda_{1} F^{\prime \prime } F^{\prime \prime \prime } - \frac{{\left( {1 - phi_{1} } \right)^{ - 2.5} M^{2} \left( {1 - phi_{2} } \right)^{ - 2.5} }}{{\left( {1 - phi_{3} } \right)^{2.5} \left( {1 + B_{e} B_{i} } \right)^{2} + \left( {B_{e} } \right)^{2} }}\left[ {\left( {1 + \beta_{e} \beta_{i} } \right)F^{\prime } + 2\beta_{e} G} \right]$$

18 $$\begin{gathered} G^{\prime \prime } - \beta - \frac{{\left( {1 - phi_{1} } \right)^{ - 2.5} M^{2} \left( {1 - phi_{2} } \right)^{ - 2.5} }}{{\left( {1 - phi_{3} } \right)^{2.5} \left( {1 + B_{e} B_{i} } \right)^{2} + \left( {B_{e} } \right)^{2} }}\left[ {\left( {1 + \beta_{e} \beta_{i} } \right)G - \frac{1}{2}\beta_{e} F^{\prime } } \right] \hfill \\ + \frac{{\nu_{f} }}{{\nu_{thnf} }}\left( {GF^{\prime } - F^{\prime } G^{\prime } } \right) + \lambda_{1} G^{\prime } G^{\prime \prime } = 0, \hfill \\ \end{gathered}$$

19 $$\left( {1 + \frac{4}{{3n_{r} }}} \right)\theta^{\prime \prime } + \frac{{K_{f} }}{{k_{thnf} }}\frac{{\left( {\rho C_{p} } \right)_{thnf} }}{{\left( {\rho C_{p} } \right)_{f} }}Pr\left( {\frac{1}{2}F^{\prime } \theta - F\theta^{\prime } } \right) + \frac{{k_{f} }}{{k_{thnf} }}PrH_{t} \theta = 0.$$

Boundary conditions in term of dimensionless form are

20 $$F\left( 0 \right) = 0, F^{\prime}\left( \infty \right) = 0, G\left( 0 \right) = 1, G\left( \infty \right) = 0, \theta \left( \infty \right) = 0, F\left( 0 \right) = 0, \theta \left( 0 \right) = 1.$$

Flow rate in term of hybrid nanoparticles^28–31^ is derived as

21 $$Cf\left( {Re} \right)^{\frac{1}{2}} = \left( {1 + \beta } \right)\left( {1 + \frac{{\Gamma }}{2}\frac{\partial U}{{\partial Y}}} \right)\frac{\partial U}{{\partial Y}} = \left( {1 + \beta } \right)\left( {F^{\prime \prime } \left( 0 \right) + \frac{\lambda }{2}\left( {F^{\prime \prime } \left( 0 \right)} \right)^{2} } \right),$$

22 $$Cg\left( {Re} \right)^{\frac{1}{2}} = \left( {1 + \beta } \right)\left( {1 + \frac{{\Gamma }}{2}\frac{\partial V}{{\partial Y}}} \right)\frac{\partial V}{{\partial Y}} = \left( {1 + \beta } \right)\left( {G^{\prime } \left( 0 \right) + \frac{\lambda }{2}\left( {G^{\prime } \left( 0 \right)} \right)^{2} } \right).$$

Heat transfer rate is formulated^13^ as

23 $$Nu\left( {Re} \right)^{{ - \frac{1}{2}}} = \frac{{K_{Thnf} }}{{K_{f} }}{\uptheta }^{\prime } \left( 0 \right).$$

## Numerical technique

**Figure 2:**
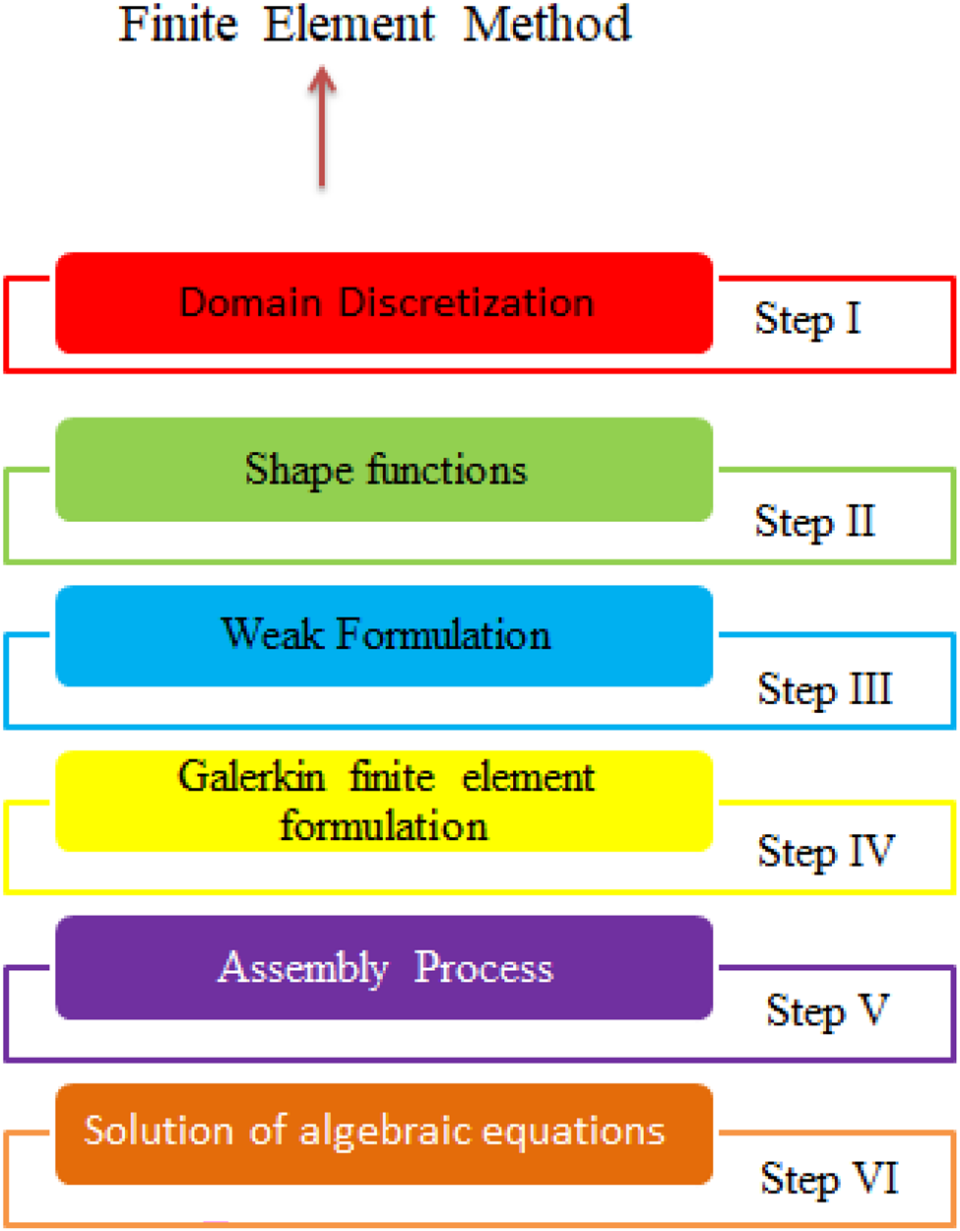
FEM related steps.

A strong numerical approach based on FEM is embraced to find results of ODEs along with boundary conditions. The methodology is explained through Fig. 2. A FEM is used to conduct the solutions of various CFD problems. It is noticed that code regarding finite element method is designed on MAPLE 18. Moreover, Maple is known as numerical and symobolic computing enviroment. It deals with numberious areas, such as numerical analysis, data processing, symobilc mathematics and visulizations. Several advantages of FEM are mentioned below.

- FEM has ability to tackle various kinds of boundary conditions for the problem arising in the modeling of different physical system in engineering doamin;
- It has also ability to simulate various types complex geometries;
- It can be easily discretization’s of derivatives very well;
- It needs low investment resources and time in term of handing problems;
- Several physical problems regarding recent developments in applied sciences are solved by FEM.

*Step I* Domain Discretization

First step is about domain discretization of problem domain. Domain is broken into small elements up to 300 elements. 300 elements are enough to simulate solution of current analysis, which is shown in Table 2. It is noticed that a system of ODEs is called strong form whereas weak form is achieved via residual method. The residuals [30 and 31] are derived as

24 $$\int \limits_{{\eta_{e} }}^{{\eta_{e + 1} }} w_{1} \left[ {F^{\prime} - H} \right]d\eta = 0,$$

25 $$\int \limits_{{\eta_{e} }}^{{\eta_{e + 1} }} w_{2} \left[ {\begin{array}{*{20}c} {\beta \left( {F^{2} H^{\prime \prime } - 2FHH^{\prime } } \right) + \frac{{\nu_{f} }}{{\nu_{thnf} }}\left( {\frac{1}{2}H^{2} - FH^{\prime } - 2G^{2} - 2\lambda \theta } \right)} \\ { + \frac{{\left( {1 - phi_{1} } \right)^{ - 2.5} M^{2} \left( {1 - phi_{2} } \right)^{ - 2.5} }}{{\left( {1 - phi_{3} } \right)^{2.5} \left( {1 + B_{e} B_{i} } \right)^{2} + \left( {B_{e} } \right)^{2} }}\left[ {\left( {1 + \beta_{e} \beta_{i} } \right)H + 2\beta_{e} G} \right]} \\ \end{array} } \right]d\eta = 0,$$

26 $$\int \limits_{{\eta_{e} }}^{{\eta_{e + 1} }} w_{3} \left[ {\begin{array}{*{20}c} {\beta \left( {G^{2} G^{\prime \prime } - 2GGG^{\prime } } \right) + + \frac{{\nu_{f} }}{{\nu_{thnf} }}\left( {GH - HG^{\prime } } \right) + \lambda_{1} G^{\prime } G^{\prime \prime } } \\ { - \frac{{\left( {1 - phi_{1} } \right)^{ - 2.5} M^{2} \left( {1 - phi_{2} } \right)^{ - 2.5} }}{{\left( {1 - phi_{3} } \right)^{2.5} \left( {1 + B_{e} B_{i} } \right)^{2} + \left( {B_{e} } \right)^{2} }}\left[ {\left( {1 + \beta_{e} \beta_{i} } \right)G - \frac{1}{2}\beta_{e} H} \right]} \\ \end{array} } \right]d\eta = 0,$$

27 $$\int \limits_{{\eta_{e} }}^{{\eta_{e + 1} }} w_{4} \left[ {\left( {1 + \frac{4}{{3n_{r} }}} \right)\theta^{\prime \prime } + \frac{{K_{f} }}{{k_{thnf} }}\frac{{\left( {\rho C_{p} } \right)_{thnf} }}{{\left( {\rho C_{p} } \right)_{f} }}Pr\left( {\frac{1}{2}H\theta - F\theta^{\prime } } \right) + \frac{{k_{f} }}{{k_{thnf} }}PrH_{t} \theta } \right]$$

**Table 2:** Simulations of $$F^{\prime } \left( {\left( {\frac{{\eta_{Max} }}{2}} \right)} \right)$$$$, G\left(\frac{{\eta }_{Max}}{2}\right)$$ and $$\theta \left(\frac{{\eta }_{Max}}{2}\right)$$ in term of mesh free study.

Number of elements	$F^{\prime } \left( {\frac{{\eta_{Max} }}{2}} \right)$	$G\left(\frac{{\eta }_{Max}}{2}\right)$	$\theta \left(\frac{{\eta }_{Max}}{2}\right)$
30	0.7034699538	0.0001271156631	0.6643453360
60	0.6654719170	0.6476105404	0.0001681814168
90	0.6528811600	0.6419911471	0.0001753839054
120	0.6466025942	0.6391737776	0.0001776462284
150	0.6428421432	0.6374817686	0.0001785704232
180	0.6403366926	0.6363523416	0.0001790046029
210	0.6385486335	0.6355451067	0.0001792273544
240	0.6372087906	0.6349398323	0.0001793468912
270	0.6344678686	0.6344678686	0.0001794104423
300	0.6353326192	0.6340911021	0.0001794425719

*Step II* Selection of Shape Function

A significant role of shape functions are used to obtain approximation solution of current analysis. Various types of shape functions are used in finite element procedure. In this procedure, linear kind of shape functions is used. Desired form of shape functions are defined as

28 $$\psi_{i} = \left( { - 1} \right)^{i - 1} \left( {\frac{{ - \eta + \eta_{i - 1} }}{{ - \eta_{i} + \eta_{i + 1} }}} \right), i = 1, 2.$$

*Step III* Weak Formulation

Equations (12)–(14) are known as strong form along with boundary conditions. In this procedure, weak forms are needed to achieve approximation solution. Collection of all terms are placed on one side and integrating it over 300 elements as presented in Table 2.

*Step IV* Finite Element Formulation

In this step, stiffness elements are obtained of current problem. Finally, global stiffness matrices are achieved over each element.

29 $$K_{ij}^{11} = \int \limits_{{\eta_{e} }}^{{\eta_{e + 1} }} \left( {\frac{{d\psi_{j} }}{d\eta }\psi_{i} } \right)d\eta , K_{ij}^{14} = 0, K_{ij}^{12} = \int \limits_{{\eta_{e} }}^{{\eta_{e + 1} }} \left( {\psi_{j} \psi_{i} } \right)d\eta , b_{i}^{1} = 0,K_{ij}^{13} = 0,$$

30 $${K}_{ij}^{22}={\int }_{{\eta }_{e}}^{{\eta }_{e+1}}\left[\begin{array}{l}-\left(\beta {\overline{F} }^{2}+{\lambda }_{1}\overline{{H }^{^{\prime}}}\right)\frac{d{\psi }_{j}}{d\eta }\frac{d{\psi }_{i}}{d\eta }-\beta 2\overline{F }\overline{H}\frac{d{\psi }_{j}}{d\eta }{\psi }_{i}+\frac{{\nu }_{f}}{{\nu }_{thnf}}\frac{1}{2}\overline{H}{\psi }_{j}{\psi }_{i}\\ \frac{{\left(1-{phi}_{1}\right)}^{-2.5}{M}^{2}{\left(1-{phi}_{2}\right)}^{-2.5}}{{{\left(1-{phi}_{3}\right)}^{2.5}\left(1+{B}_{e}{B}_{i}\right)}^{2}+{\left({B}_{e}\right)}^{2}}\left[\left(1+{\beta }_{e}{\beta }_{i}\right){\psi }_{j}{\psi }_{i}\right]\\ -\frac{{\nu }_{f}}{{\nu }_{thnf}}\left(\overline{F }\right)\frac{d{\psi }_{j}}{d\eta }{\psi }_{i}\end{array}\right]d\eta ,{b}_{i}^{4}=0,$$

31 $${K}_{ij}^{23}={\int }_{{\eta }_{e}}^{{\eta }_{e+1}}\left[\frac{{\left(1-{phi}_{1}\right)}^{-2.5}{M}^{2}{\left(1-{phi}_{2}\right)}^{-2.5}}{{{\left(1-{phi}_{3}\right)}^{2.5}\left(1+{B}_{e}{B}_{i}\right)}^{2}+{\left({B}_{e}\right)}^{2}}2{\beta }_{e}{\psi }_{j}{\psi }_{i}-\frac{{2\nu }_{f}}{{\nu }_{thnf}}\overline{G}{\psi }_{j}{\psi }_{i}\right]d\eta ,$$

32 $${K}_{ij}^{24}={\int }_{{\eta }_{e}}^{{\eta }_{e+1}}-\left[\frac{\lambda {2\nu }_{f}}{{\nu }_{thnf}}{\psi }_{j}{\psi }_{i}\right]d\eta , {K}_{ij}^{21}=0, {b}_{i}^{2}=0, {K}_{ij}^{31}=0,{K}_{ij}^{34}=0, {K}_{ij}^{41}=0,$$

33 $${K}_{ij}^{33}={\int }_{{\eta }_{e}}^{{\eta }_{e+1}}\left[\begin{array}{l}-\beta \left(\overline{G }\overline{G }+{\lambda }_{1}\overline{{G }^{^{\prime}}}\right)\frac{d{\psi }_{j}}{d\eta }\frac{d{\psi }_{i}}{d\eta }-\beta \overline{G }\overline{G}\frac{d{\psi }_{j}}{d\eta }{\psi }_{i}+\frac{{\nu }_{f}}{{\nu }_{thnf}}\overline{G}\frac{d{\psi }_{j}}{d\eta }{\psi }_{i}\\ -\frac{{\left(1-{phi}_{1}\right)}^{-2.5}{M}^{2}{\left(1-{phi}_{2}\right)}^{-2.5}}{{{\left(1-{phi}_{3}\right)}^{2.5}\left(1+{B}_{e}{B}_{i}\right)}^{2}+{\left({B}_{e}\right)}^{2}}\left(1+{\beta }_{e}{\beta }_{i}\right){\psi }_{j}{\psi }_{i}\\ +\frac{{\nu }_{f}}{{\nu }_{thnf}}\overline{G}\frac{d{\psi }_{j}}{d\eta }{\psi }_{i}-\frac{{\nu }_{f}}{{\nu }_{thnf}}\overline{H}\frac{d{\psi }_{j}}{d\eta }{\psi }_{i}\end{array}\right]d\eta ,$$

34 $$K_{ij}^{32} = \int \limits_{{\eta_{e} }}^{{\eta_{e + 1} }} \left[ {\frac{{\left( {1 - phi_{1} } \right)^{ - 2.5} M^{2} \left( {1 - phi_{2} } \right)^{ - 2.5} }}{{2\left( {1 - phi_{3} } \right)^{2.5} \left( {1 + B_{e} B_{i} } \right)^{2} + \left( {B_{e} } \right)^{2} }}\beta_{e} \psi_{j} \psi_{i} } \right]d\eta , b_{i}^{3} = 0, K_{ij}^{42} = 0,$$

35 $$K_{ij}^{44} = \int \limits_{{\eta_{e} }}^{{\eta_{e + 1} }} \left[ {\begin{array}{*{20}l} { - \left( {1 + \frac{4}{{3n_{r} }}} \right)\frac{{d\psi_{j} }}{d\eta }\frac{{d\psi_{i} }}{d\eta } + \frac{{K_{f} }}{{k_{thnf} }}\frac{{\left( {\rho C_{p} } \right)_{thnf} }}{{\left( {\rho C_{p} } \right)_{f} }}Pr\left( {\frac{1}{2}\overline{H}\psi_{j} \psi_{i} } \right)} \\ { - \frac{{K_{f} }}{{k_{thnf} }}\frac{{\left( {\rho C_{p} } \right)_{thnf} }}{{\left( {\rho C_{p} } \right)_{f} }}\overline{F}\frac{{d\psi_{j} }}{d\eta }\psi_{i} + \frac{{k_{f} }}{{k_{thnf} }}PrH_{t} \psi_{j} \psi_{i} } \\ \end{array} } \right]d\eta ,K_{ij}^{43} = 0.$$

*Step V* Assembly Process

Assembly process is an integral part of finite element method. Stiffness matrices are formulated using concept of assembly approach.

*Step VI* Solution of Algebraic Equations

In final step, algebraic equations are achieved under visualized tolerance (${10}^{-5}$) which is defined as

36 $$\left| {\frac{{\aleph_{i + 1} - \aleph_{i} }}{{\aleph^{i} }}} \right| < 10^{ - 5} .$$

### Validatio of numerical study

Table 3 is prepared to visulized the validation of numerical study with published work. It is noticed that good agreement among pubish work^19^ and present study by disppareing impacts tri-hybrid nanoparticles and non-Newtonin behaviour. These comaraive simulations are recorded in Table 3.

**Table 3:** Validation of numerical simulations in term of skin friction coefficients and heat transfer rate with published work^19^ when ${ph}_{1}={ph}_{2}={ph}_{3}=0, {\beta }_{e}=0, Pr=0.7,\beta =0$ and ${\lambda }_{1}=0.$

$\lambda$	Malik et al.^19^			Present simulations		
	$-Cf{\left(Re\right)}^\frac{1}{2}$	$-Cg{\left(Re\right)}^\frac{1}{2}$	$-Nu{\left(Re\right)}^{-\frac{1}{2}}$	$-Cf{\left(Re\right)}^\frac{1}{2}$	$-Cg{\left(Re\right)}^\frac{1}{2}$	$-Nu{\left(Re\right)}^{-\frac{1}{2}}$
0.0	1.0253	0.6153	0.4295	1.0251320192	0.61529943250	0.4294719073
1.0	2.2007	0.8492	0.6121	2.2007397320	0.84977941223	0.6126020922
10	8.5041	1.3990	1.0097	8.5046102402	1.3995234132	1.0097023340

### Grid independent investigation

The code regarding finite element method is designed in MAPLE 18. The code is varified with already published works. The numerical reuslts of grid indepenet analaysis are recoreded in Table 2. This table reveals convergence investiagation for 300 elements. It is estimated that results are recoreded in table versus incresing number of elements at mid of each temeprature and vecloity profile. Hence, solution of problemes is converged by taking 300 elements whreas graphical and numerical results are simulated by taking 300 elements. Comparative study is prented in Table 3.

## Outcomes and discussion

The formulation of 3D model is developed in presence of Williamson fluid and Maxwell fluid. Dynamics behavior of tri-hybrid nanoparticles is implemented under ion slip and Hall currents. Heat source and thermal radiation are added into heat energy equation. The correlations in view of tri-hybrid nanomaterial along with thermal properties are added. Finite element approach is utilized to obtain numerical consequences. The combine study of Williamson and Maxwell liquids is analyzed. Explanations of graphical results are addressed below.

### Dynamics analysis regarding velocity fields

**Figure 3:**
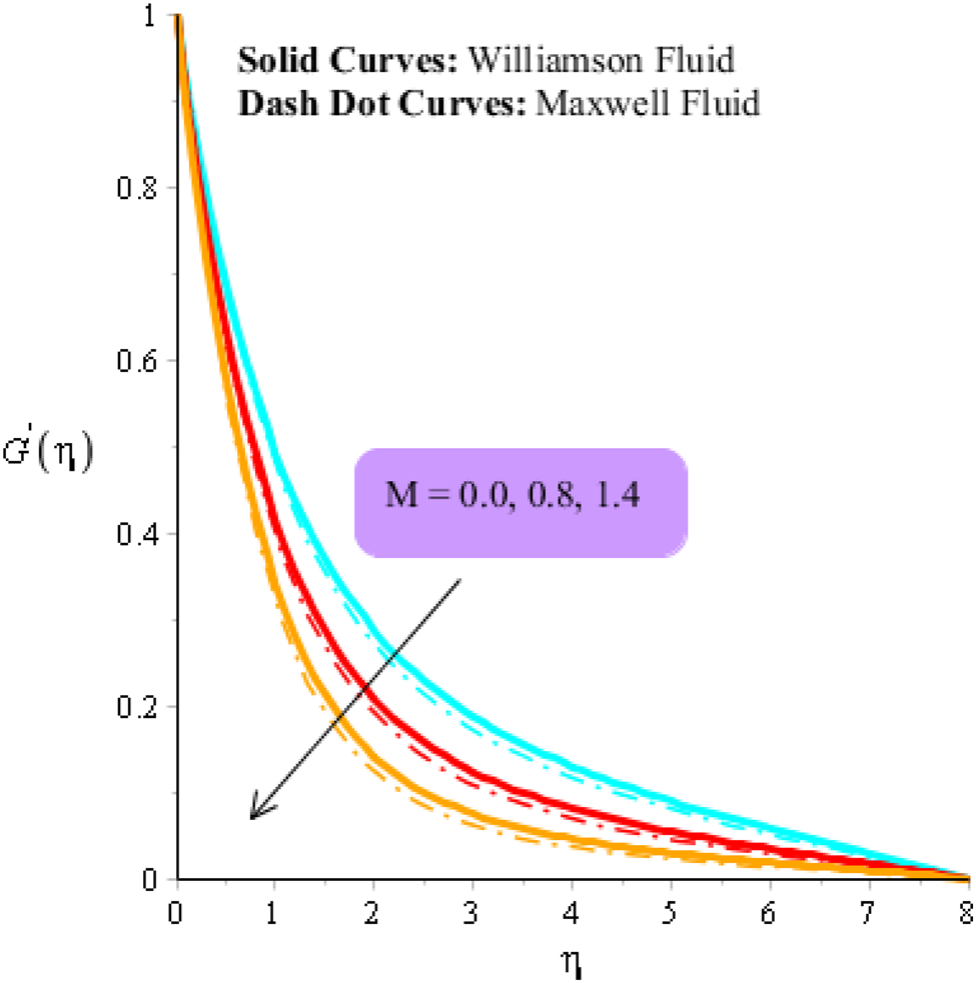
Influence of $M$ versus secondary velocity filed.

**Figure 4:**
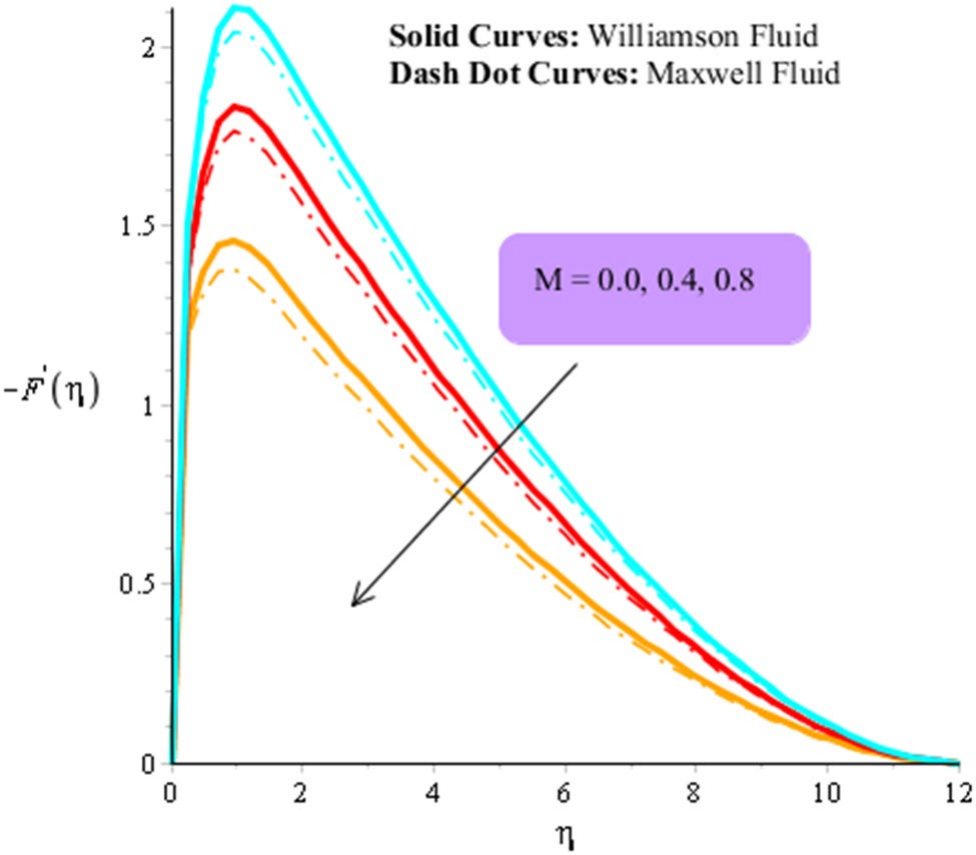
Influence of $M$ versus primary velocity filed.

**Figure 5:**
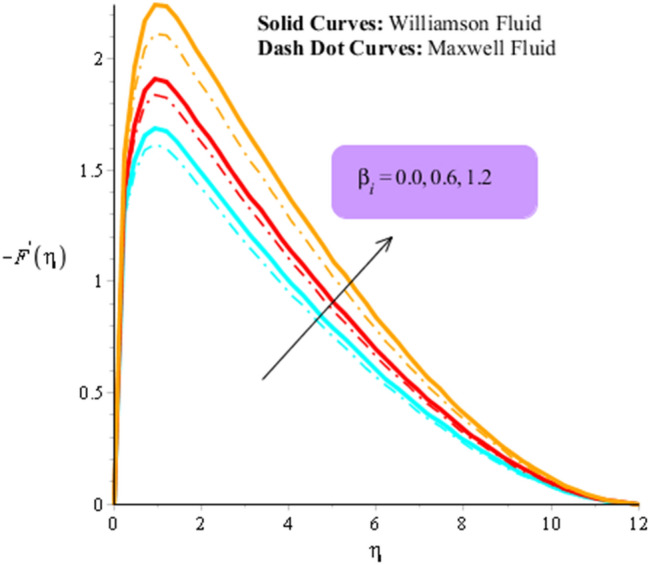
Influence of ${\beta }_{i}$ versus primary velocity filed.

**Figure 6:**
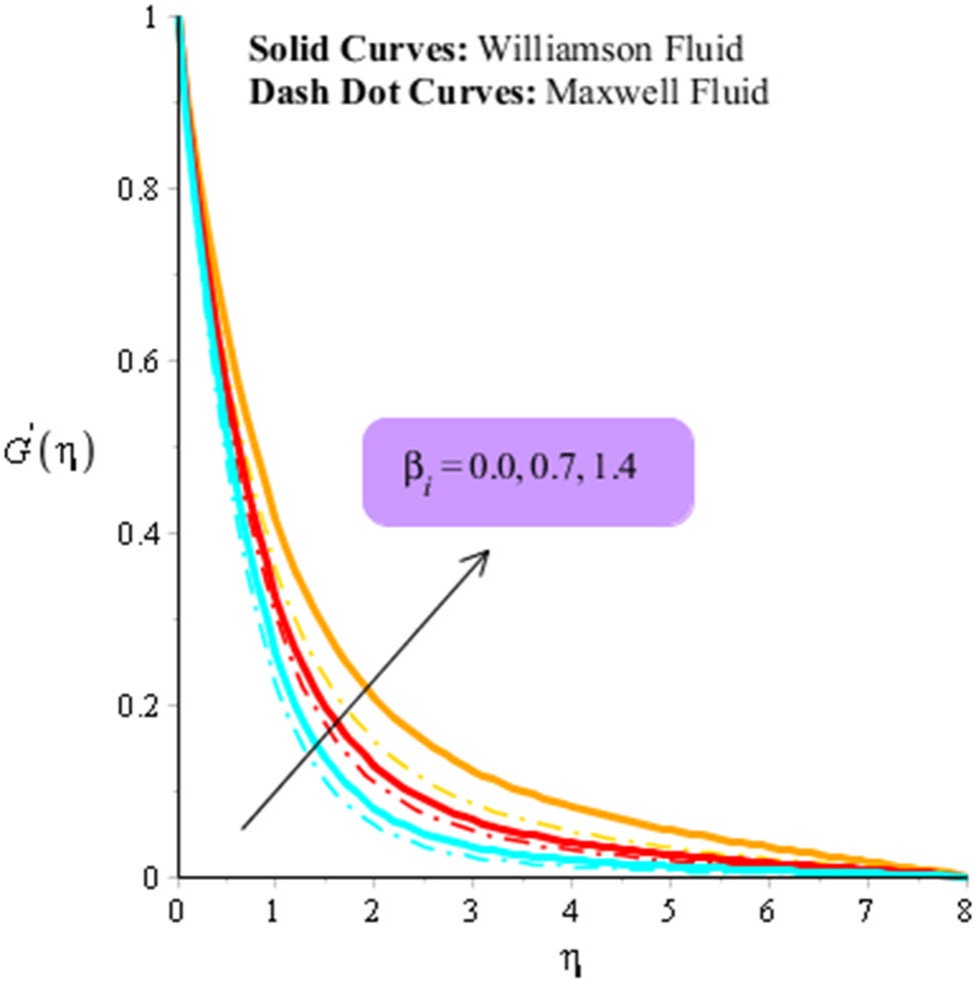
Influence of ${\beta }_{i}$ versus secondary velocity filed.

**Figure 7:**
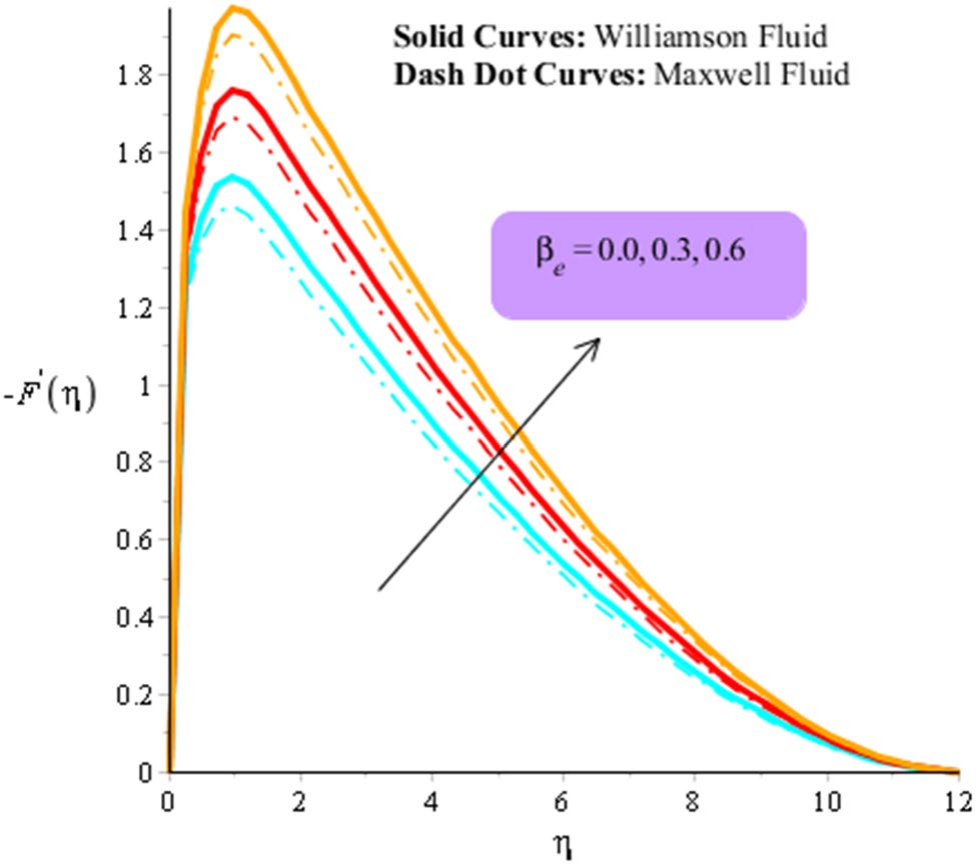
Influence of ${\beta }_{e}$ versus primary velocity filed.

**Figure 8:**
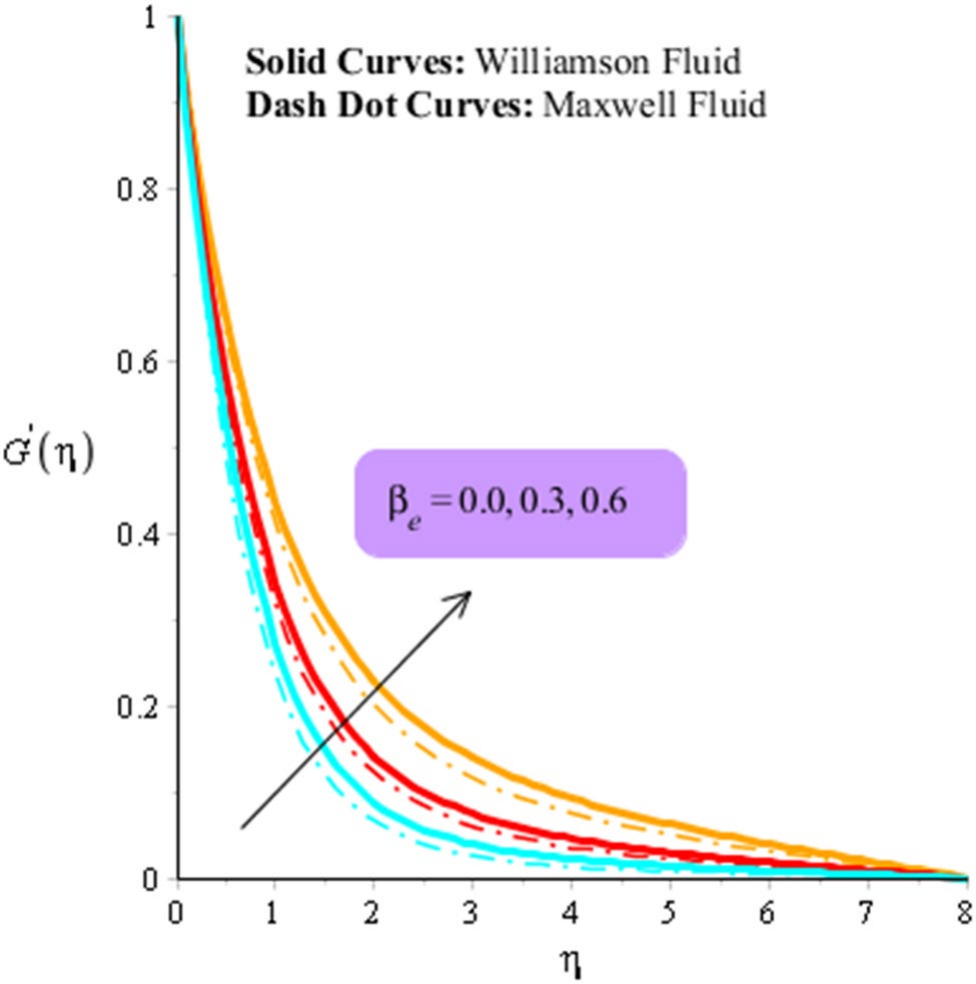
Influence of ${\beta }_{e}$ versus secondary velocity filed.

Figures 3, 4, 5, 6, 7 and 8 are plotted to visualize comparative investigation among Maxwell fluid and Williamson fluid considering impact of Hall parameter, ion slip number, magnetic number and bouncy parameter on velocity field. It is noticed that solid curves are captured for behavior of Williamson liquid while dot curves are prepared for visualization of Maxwell fluid. Figures 3 and 4 is plotted to determine impact of magnetic parameter on velocity field in term of y- and x-directions. From this figure, it is estimated that motion regarding fluid particles is induced using wall velocity. However, impact of Lorentz force creates resistance among fluid particles using implication of magnetic parameter. The flow regarding fluidic particles becomes slow down when magnetic field is implemented. Lorentz force is produced using appearance of magnetic field. Flow is slow down because of negative Lorentz force is implemented. Additionally, magnetic parameter is formulated using concept of Lorentz force in momentum equations. Magnetic parameter is inserted along z-direction regarding fluidic motion. Due to opposite direction of flow and magnetic field, flow becomes slow down. Thickness regarding momentum layers is decreased when magnetic parameter is increased. Moreover, flow for case of appetence of magnetic field is higher than flow for the case of disappearance of magnetic field. Figures 5, 6, 7 and 8 are sketched to determine observation of ion slip and Hall parameters on motion regarding fluid particles in term of y- and x-directions. The acceleration is enhanced when ion slip and Hall parameters are increased. This is because ion slip and Hall parameters are appeared in generalized Ohm’s. Therefore, velocity of fluidic particles is increased. Moreover, it visualized that thickened based on momentum layers are higher for the cade Williamson fluid than as compared thickness for the case of Maxwell liquid. It is noticed that Lorentz force and magnetic parameter are declined when ion slip and Hall parameters are enhanced. The reduction of Lorentz force makes enhancement into fluidic motion. Further, ${\beta }_{e}$ and ${\beta }_{i}$ are appeared in denominator in momentum equations. Hence, inversely proportion relation is appeared versus Lorentz force. Therefore, flow in both directions is increased when ${\beta }_{e}$ and ${\beta }_{i}$ are increased. Thickness of momentum layers are decreased versus enhancement in ${\beta }_{e}$ and ${\beta }_{i}.$ The flow for case of disappearance of ${\beta }_{e}$ and ${\beta }_{i}$ is higher than flow for the case of appurtenance of ion slip and Hall parameters.

### Dynamics analysis regarding heat energy

**Figure 9:**
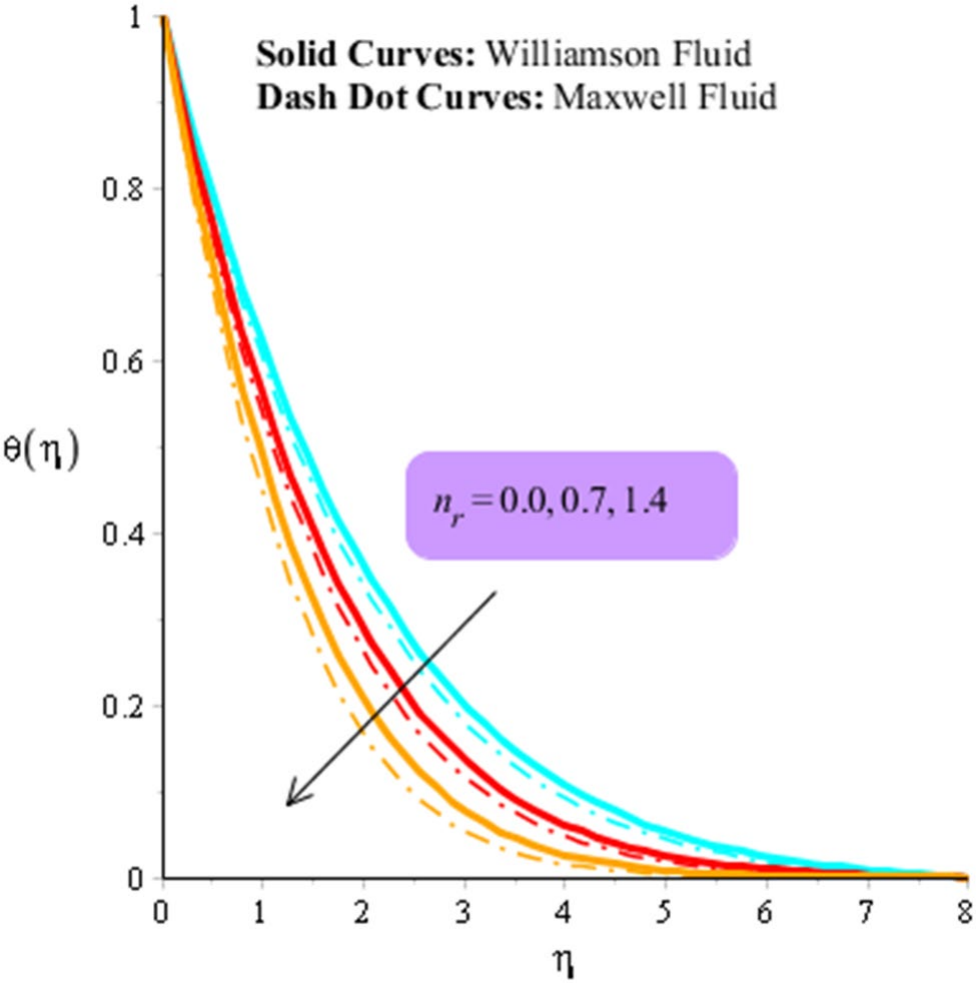
Influence of ${n}_{r}$ versus temperature filed.

**Figure 10:**
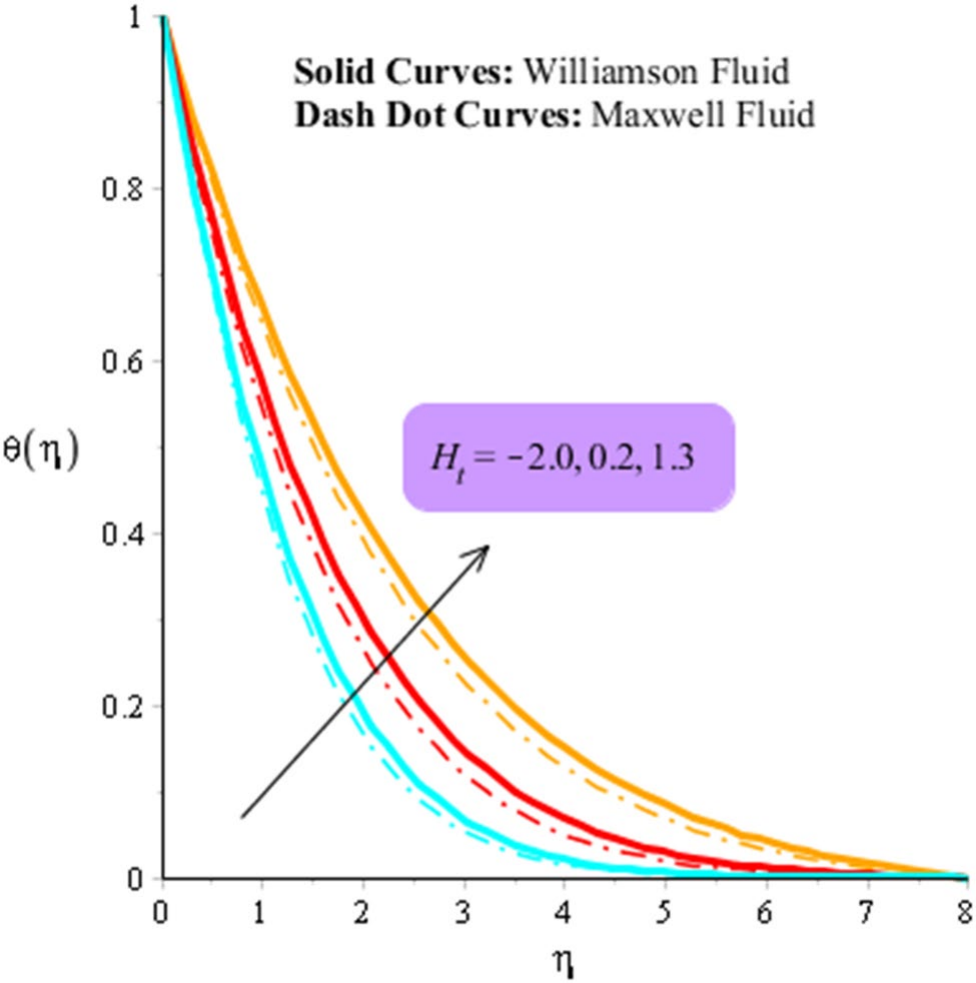
Influence of ${H}_{t}$ versus temperature filed.

**Figure 11:**
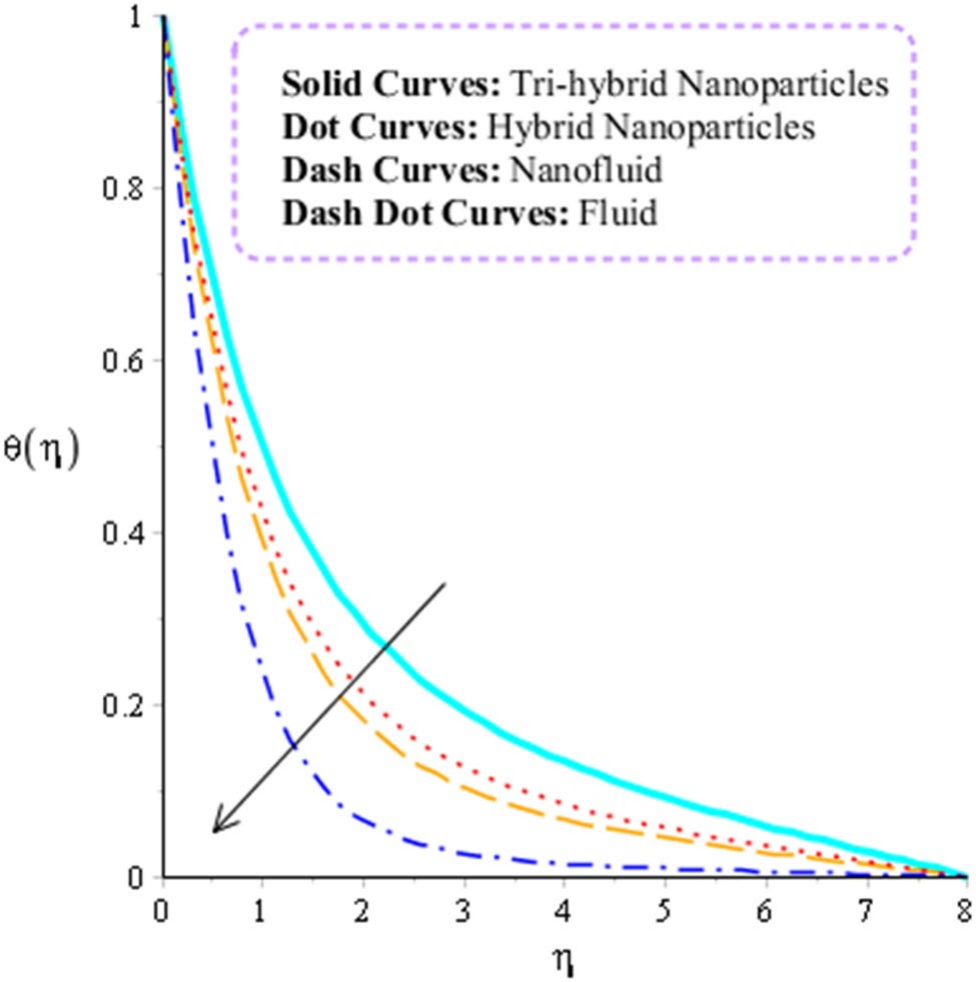
Comparison heat energy performance for tri-hybrid nanoparticles, nanofluid and fluid and hybrid nanoparticles.

Figures 9 and 10 are plotted to observe the behavior of thermal radiation number and heat source number on heat energy. It is estimated that comparative simulations among Williamson liquid and Maxwell fluid on heat energy is measured inserting ternary hybrid nanoparticles. Figure 11 captures comparison study into fluid, nanoparticles, nanofluid and hybrid nanofluid. The role radiation parameter on temperature curves are carried out by Fig. 9. From this figure, it is investigated that the production related to heat energy is reduced when radiation parameter is enhanced. It is because heat energy is transferred in term of away from the wall due to thermal radiation ways. Therefore, amount regarding thermal energy is reduced. Moreover, production related to thermal energy for the case of Williamson fluid is higher that production based on heat energy for the case of Maxwell fluid. Physically, heat energy moves away from the surface of rotating cone due to thermal radiation. Therefore, heat energy is reduced when thermal radiation number is increased. It is noticed that inverse proportional relation is observed versus impact of thermal radiation. An increment in thermal radiation number brings declination into heat energy. Thermal layers thickness of boundary layers is decreased when thermal radiation number is increased. Figure 10 predicts investigation of heat source parameter on heat energy. Heat energy is boosted when heat source parameter is increased. This is because heat source is implemented at wall via surface of sheet. Heat energy can be easily managed using heat source parameter. The impacts of two kinds impacts regarding ${H}_{t}$ are discussed which are based on heat source and heat generation phenomena’s. It is noticed that positive numerical values of ${H}_{t}$ are implemented for heat generation and magnetite numerical values of ${H}_{t}$ are implemented for heat absorption. Thickness related to thermal layers can be managed by variation in heat source parameter. Moreover, production related to thermal energy for the case of Williamson fluid is higher that production based on heat energy for the case of Maxwell fluid. Figure 11 illustrates visualizations of thermal enhancement inserting fluid, hybrid nanofluid, nanofluid and tri-hybrid nanofluid. It is noticed that solid curves are made by tri-hybrid nanofluid and dot curves are drawn for apprence of hybrid nanoparticles. Further, dash and dot dash curves are captured for fluid and nanofluid. From this figure, it is investigated that tri-hybrid nanoparticles are significant to obtain maximum amount regarding heat energy rather than hybrid nanoparticles, fluid and nanofluid.

### Dynamics analysis regarding Nusselt number, divergent velocities and heat transfer rate

Table 4 demonstrates numerical effects of various (defined parameters) on velocity gradients and Nusselt number. From Table 4, it is studied that Nusselt number is reduced versus heat source, magnetic parameter but heat transfer rate is boosted when ion slip and Hall parameters are increased. It is noticed that divergent velocities are known as skin friction coefficients. The surface forces per unit area are termed as skin friction coefficients. Skin friction coefficients are declined versus impacts of ion slip and Hall parameters. Flow rates in term of vertical and horizontal directions become slow down versus increment values of Hall parameter and ion slip number. Heat source number produces declination into flow rates in term of vertical and horizontal directions. Comparative simulations among Williamson fluid and Maxwell fluid against change in magnetic number and Prandtl number are recorded in Table 5. It is estimated that heat transfer rate is enhanced versus variation in Prandtl number but heat transfer rate is declined against change in magnetic parameter. Additionally, production for heat transfer rate for case of Williamson liquid is higher than heat transfer rate for case of Maxwell fluid.

**Table 4:** Numerical effects of flow rates and Nusselt number versus ${\beta }_{e}, M, {\beta }_{i}$ and ${H}_{t}.$

Change in parameters		$-Cf{\left(Re\right)}^\frac{1}{2}$	$-Cg{\left(Re\right)}^\frac{1}{2}$	$-Nu{\left(Re\right)}^{-\frac{1}{2}}$
	0.0	0.5553479204	1.748606357	0.7829787333
$M$	0.4	0.5753441468	1.778320364	0.7530277233
	0.8	0.5852129658	1.796435697	0.7251228524
	0.0	0.5553079541	1.680368431	0.7799653389
${\beta }_{e}$	0.2	0.5350949743	1.502998494	0.7827366919
	0.4	0.5250832751	1.409744755	0.7920984199
	0.0	0.5551030346	1.727216716	0.7784422264
${\beta }_{i}$	0.8	0.5351498653	1.716458189	0.7866359920
	1.4	0.5151749487	1.709309779	0.7959683439
	-1.3	0.5162153062	1.652216728	0.3736565052
${H}_{t}$	0.4	0.4313655409	1.611410548	0.248580176
	1.5	0.4146696632	1.592119492	0.083446514

**Table 5:** Comparative numerical performance among Williamson fluid and Maxwell fluid versus impacts of magnetic parameter and Prandtl number.

		Maxwell fluid	Williamson fluid
		$-Nu{\left(Re\right)}^{-\frac{1}{2}}$	$-Nu{\left(Re\right)}^{-\frac{1}{2}}$
	0.0	0.7829754254	1.498735588
$M$	0.4	0.7837240327	1.619323451
	0.8	0.7861334864	1.739324158
	206	0.3562296680	1.389662075
$Pr$	208	0.4278509806	1.389671389
	210	0.9917767611	1.389693808

## Conclusions and important findings

Mathematical model regarding two non-Newtonian fluids (Maxwell and Williamson fluids) is developed in the presence of ion-slip and Hall currents over a cone. Tri-hybrid nanoparticles are implemented in ethylene glycol called base fluid. Main observations of problem are listed below.

- 300 elements regarding problem domain are confirmed to discretize problem domain;
- Thermal performance as well as flow performance for the case Williamson fluid is better than for case of Maxwell fluid;
- Production via thermal energy is boosted when heat source parameter is enhanced;
- Radiation parameter declines heat energy performance as well as thickness regarding thermal boundary layers;
- Ion slip and Hall parameters arguments flow into fluid particles;
- Maximum heat energy is produced for tri-hybrid nanoparticles rather than fluid, hybrid nanofluid and nanoparticles;
- Production for heat transfer rate for case of Williamson liquid is higher than heat transfer rate for case of Maxwell fluid;
- Significant achievement in thermal enhancement by using mixtures of tri-hybrid nanoparticles which are applicable in coolants in automobile, fluidic dynamics, production of solar energy, engineering process, cancer therapy, hair care products and electrical insulators.

## Data Availability

The datasets generated/produced during and/or analyzed during the current study/research are available from the corresponding author on reasonable request.

## List of symbols

$$y,x,z$$: Space coordinates (m)
${B}_{0}$: Magnitude of magnetic field (Akgs^−2^)
$thnf$: Tri-hybrid nanoparticles
$$\nu$$: Kinematic viscosity (m^2^s^−1^)
${T}_{\infty }$: Ambient fluid temperature (k)
$$G$$: Gravitational acceleration (N)
$K$: Thermal conductivity (Wm^−1^)
$${n}_{r}$$: Thermal radiation
$$\lambda$$: Bouncy number
$\rho$: Fluid density (Kgm^−3^)
$$Nu$$: Nusselt number
$f, bf$: Fluid and base fluid
$${\lambda }_{1}, \beta$$: Fluid numbers
${\sigma }^{*}$: Stefan-Boltzmann constant
$\Omega$: Angular velocity (ms^−1^)
$${\psi }_{i}$$: Shape function
$F G, \theta$: Dimensionless velocities and temperature
$$Re$$: Reynolds number
CFD: Computational fluid dynamics
${\varvec{B}}$: Magnetic induction (weber/m^2^)
${\varvec{V}}$: Velocity vector (ms^−1^)
${\varvec{\tau}}$: Fluid tensor vector
${\varvec{E}}$: Electrical field vector (Vm^−1^)
$V, U, W$: Velocity components (ms^−1^)
$\sigma$: Electrical conductivity (Sm^−1^)
${\beta }_{e}, {\beta }_{i}$: Hall and ion slip numbers
$$\Gamma$$: Fluid number
$$T$$: Fluid temperature (k)
${phi}_{2}, {phi}_{3}, {phi}_{2}$: Volume fraction for nanoparticles
$M$: Magnetic field number
$$Pr$$: Prandtl number
${H}_{t}$: Heat source number
${c}_{p}$: Specific heat capacity (JKg^−1^K)
$$Re$$: Reynolds number
FEM: Finite element method
${Q}_{0}$: Heat generation/absorption
$$e$$: Element
$${T}_{w}$$: Wall temperature (k)
$Cf, Cg$: Skin friction coefficients
$$Nu$$: Nusselt number
FEM: Finite element method
$${w}_{1}, {w}_{2}, {w}_{3}$$: Weight functions
$$\frac{d}{dt}$$: Total derivative
${\varvec{J}}$: Current density (Am^−2^)
$$\nabla$$: Gradient operator
${\varvec{q}}$: Heat flux vector (Wm^2^)
